# Dysregulation of B7 family and its association with tumor microenvironment in uveal melanoma

**DOI:** 10.3389/fimmu.2022.1026076

**Published:** 2022-10-14

**Authors:** Yao Chen, Anfu Zheng, Yao Zhang, Mintao Xiao, Yueshui Zhao, Xu Wu, Mingxing Li, Fukuan Du, Yu Chen, Meijuan Chen, Wanping Li, Xiaobing Li, Yuhong Sun, Li Gu, Zhangang Xiao, Jing Shen

**Affiliations:** ^1^ Laboratory of Molecular Pharmacology, Department of Pharmacology, School of Pharmacy, Southwest Medical University, Luzhou, Sichuan, China; ^2^ Pidu District People’s Hospital, Chengdu, Sichuan, China; ^3^ Cell Therapy & Cell Drugs of Luzhou Key Laboratory, Luzhou, Sichuan, China; ^4^ South Sichuan Institute of Translational Medicine, Luzhou, Sichuan, China; ^5^ Department of Oncology, The Affiliated Hospital of Southwest Medical University, Luzhou, China

**Keywords:** B7 family, uveal melanoma, bioinformatics analysis, tumor immune microenvironment, Single-cell RNA sequencing

## Abstract

**Background:**

Uveal melanoma (UVM) is the most common primary intraocular malignancy in adults with a poor prognosis. B7 family is an important modulator of the immune response. However, its dysregulation and underlying molecular mechanism in UVM still remains unclear.

**Methods:**

Data were derived from TCGA and GEO databases. The prognosis was analyzed by Kaplan-Meier curve. The ESTIMATE algorithm, CIBERSORT algorithm, and TIMER database were used to demonstrate the correlation between B7 family and tumor immune microenvironment in UVM. Single-cell RNA sequencing was used to detect the expression levels of the B7 family in different cell types of UVM. UVM was classified into different types by consistent clustering. Enrichment analysis revealed downstream signaling pathways of the B7 family. The interaction between different cell types was visualized by cell chat.

**Results:**

The expression level of B7 family in UVM was significantly dysregulated and negatively correlated with methylation level. The expression of B7 family was associated with prognosis and immune infiltration, and B7 family plays an important role in the tumor microenvironment (TME). B7 family members were highly expressed in monocytes/macrophages of UVM compared with other cell types. Immune response and visual perception were the main functions affected by B7 family. The result of cell chat showed that the interaction between photoreceptor cells and immune-related cells was mainly generated by HLA-C-CD8A. CABP4, KCNJ10 and RORB had the strongest correlation with HLA-C-CD8A, and their high expression was significantly correlated with poor prognosis. CABP4 and RORB were specifically expressed in photoreceptor cells.

**Conclusions:**

Dysregulation of the B7 family in UVM is associated with poor prognosis and affects the tumor immune microenvironment. CABP4 and RORB can serve as potential therapeutic targets for UVM, which can be regulated by the B7 family to affect the visual perception and immune response function of the eye, thus influencing the prognosis of UVM.

## Introduction

Uveal melanoma (UVM), as a rare disease, occurs mainly in Caucasians and is the most common primary intraocular tumor in adults (1). The most common sites of UVM are choroid in 8033 cases, iris 285 (4%), ciliary body 492 (6%), and choroid 7256 (90%) (2). White skin, eyes with light color, skin or iris or choroid nevus, and mutations in *BRCA1* associated protein 1 are all the host predisposing factors for UVM (3). But the complex pathogenic mechanism still remains unclear. UVM has a high tendency to rotate migration, leading to high mortality, poor long-term prognosis and more than 50% of deaths (4). The basic treatment methods include surgical treatment, radiotherapy and chemotherapy. Though effective, nearly 50% of patients still develop metastatic disease, and the current treatment of patients with metastasis is still poor (5). Immunotherapy, as a new cancer treatment, has shown encouraging results in clinical trials and may become a major treatment option in many cancers over the next decade (6). However, the clinical benefit of immunotherapy in UVM is limited (7). Thus, we aimed to better understand the immunological features of UVM and pave the way for designing successful immunotherapy for this disease.

B7 family, as co-stimulatory or co-suppressive molecules of immunity, not only provides critical positive signals to stimulate and support the role of T cells but also provides negative signals to control and inhibit T cell responses (8). The growing B7 family now consists of 10 members: *B7-1, B7-2, B7-DC, B7-H1, B7-H2, B7-H3, B7-H4, B7-H5, B7-H6, B7-H7*. Each member contains at least 15% of the binding amino acid sequence expressed by antigen-presenting cells (APC) or tumor cells (9). Current studies have concluded that B7 family is dysregulated in multiple cancers and has implications for cancer infiltration, metastatic potential and prognosis. Thus, they can be used as new cancer biomarkers, such as soluble *B7-H3* and soluble *B7-H4*, which have been shown to be prognostic biomarkers in ovarian and renal cancers (10–13). Furthermore, dysregulation of B7 family affects TME. Sai Han et al. found that *B7-H3* and *B7-H4* overexpression plays a negative role in cervical cancer microenvironment (14). PD-L1/PD-1 signaling pathways regulate TME and mediate tumor escape (15). The overexpression of *B7-H2* promotes the formation of TME in colorectal cancer (16). In UVM, existing studies have found that members of the B7 family are also differentially expressed (17), the TME is affected (18), and anti-tumor immunity is positively or negatively regulated (19, 20). However, the expression patterns of each member of the B7 family are still unclear and little progress has been made in the study of their biological functions and mechanisms.

As an entity composed of multiple cell groups, tumor is characterized by high complexity and heterogeneity (21). With the development of high-throughput sequencing, there has been a major breakthrough in the analysis and understanding of tumors (22), making it possible to analyze life activities and elucidate genomes and transcriptomes at the molecular level. RNA sequencing (RNA-seq) has high resolution and coverage, which can not only quantify gene expression, but also identify alternative splicing genes, discover new transcripts and detect allele-specific expression (23). However, RNA sequencing (RNA-seq) is typically performed “in bulk” and represents an average of gene expression patterns, thus potentially hiding biologically relevant differences between cells, especially for tumors with complex heterogeneity (24). Single-cell RNA sequencing (scRNA-seq) is a powerful tool for analyzing the complexity of solid tumors, including genomics, transcriptomics, proteomics, epigenomics and metabolomics sequencing, to decipher the cellular and molecular landscape at single-cell resolution (25). The potential to differentiate gene expression at the single-cell level has transformed cancer research into a new paradigm and provided new insights into cancer evolution, tumor heterogeneity, and the TME (26).

In this study, we combined RNA-seq and scRNA-seq analyses to explore the expression levels and prognostic value of B7 family members, as well as the relationship of B7 family with the UVM immune microenvironment. We also analyzed B7 family related biological functions and signaling pathways. Moreover, potential therapeutic targets for UVM, which can be regulated by the B7 family, were determined.

## Materials and methods

### Data sources and processing

The Cancer Genome Atlas (TCGA) database (http://cancergenome.nih.gov/) was used to obtain the UVM gene expression data and clinical data. Data for 80 patient samples were obtained. Single-cell sequencing data for 3 metastatic and 8 orthotopic tumor samples was obtained from GSE139829 data set in the Gene Expression Omnibus (GEO) database. B7 family differential expression analysis data were downloaded from GSE176345 dataset in GEO database. cBioPortal (http://cbioportal.org/) is a Web resource for exploring, visualizing, and analyzing multidimensional cancer genomics data (27). In cBioPortal website, the relationship between mRNA expression level of B7 members and DNA methylation and copy number change was analyzed, and the DEGs were obtained according to the high and low expression of B7 family. Tumor immune estimate resources (TIMER) (https://cistrome.shinyapps.io/timer/) is used for the comprehensive study of the interaction between tumor and immune molecules (28). Using the TIMER website, we visualized the correlation between B7 expression, gene alteration and the level of immunologic infiltration in UVM. There is no data of B7-H6 in UVM in TIMER website.

### ESTIMATE, CIBERSORT and correlation analysis

Stromal score and immune score were obtained by limma and estimate R packages. CIBERSORT was applied to immune cell analysis by using TCGA gene expression data (29). We conducted CIBERSORT analysis through limma and GENE. CIBERSORT packages in R software. And the correlation analysis was carried out by ggcorrplot and ggthemes packages in R software. R software version is 3.6.1.

### Single cell sequencing analysis

Single-cell sequencing data from 11 samples were analyzed by the Seurat (4.1.1) package. Firstly, quality control was performed in three steps: the first step creates Seurat objects based on the uniform criteria of min.cells = 3 and min.features = 50, the second step filters the data based on nFeature_RNA > 200 and percent.mt < 10, and finally, the data are normalized using the “LogNormalize” method. 11 samples were progressively quality controlled and the respective top 2000 highly variable genes were identified based on the FindVariableFeatures function. Anchors were identified through the FindIntegrationAnchors function. Subsequently, the 11 samples were integrated by the CCA algorithm. In summary, integrated Seurat objects containing 171941 single cells were obtained. Finally, dimensionality reduction clustering was performed based on the first 20 principal components using the t-distributed stochastic neighbor. Cumulatively, 34 different cell clusters were obtained.

### CellChat analysis

To study the interactions between cells and identify the mechanism of the communicating molecules at a single-cell resolution, the R package “CellChat” (v1.1.3) was used for cells involved in 13 cell groups. A database of signaling molecular interactions exists in this package, consisting of 60% of paracrine/autocrine signaling interactions, 21% of extracellular matrix (ECM)-receptor interactions and 19% of cell-cell contact interactions. Quantification of intercellular interactions is calculated based on differential expression of ligand-receptor pairs (30).

### Consistency cluster analysis and principal component analysis

Consistency cluster analysis was performed according to the expression profile of B7 family, and the results of consistency cluster analysis were used to make a principal component analysis (PCA). Consistency cluster analysis was performed by the ConsensusClusterPlus package in R software. The heatmap was constructed by the ggplots package in R software.

### Function and pathway analysis

We divided the samples into two groups according to the high and low expression of B7 family by consistency cluster analysis. DEGs were obtained by limma R software packages. For enriched genes, the significant change in expression was determined by Log2 based ratio (μ mean altered/μ mean unaltered) (log > 2 for over-expression, log < 2 for under-expression), and the querying event results were FDR < 0.05. The genes with -log10 FDR > 1.3 and log ratio > 2 or log ratio < -2 were selected for further analysis. Finally, the DEGs were used for GO (Gene Ontology) enrichment analysis and KEGG (Kyoto Encyclopedia of Genes and Genomes) enrichment analysis of B7 family members in the DAVID function annotation tool (https://david.ncifcrf.gov). GO includes biological process, molecular function and cellular component. To obtain important metabolic process, the count and P values were considered together.

### Statistical analysis

Statistical analysis was performed by using GraphPad Prism 7. Student’s t-test was used to compare the difference of two groups and one-way ANOVA to compare multiple groups. Overall survival was shown as a Kaplan-Meier curve, which was calculated by using the log-rank test. P < 0.05 was considered statistically significant.

## Results

### The dysregulation and prognostic potential of B7 family members in UVM

**Figure 1:**
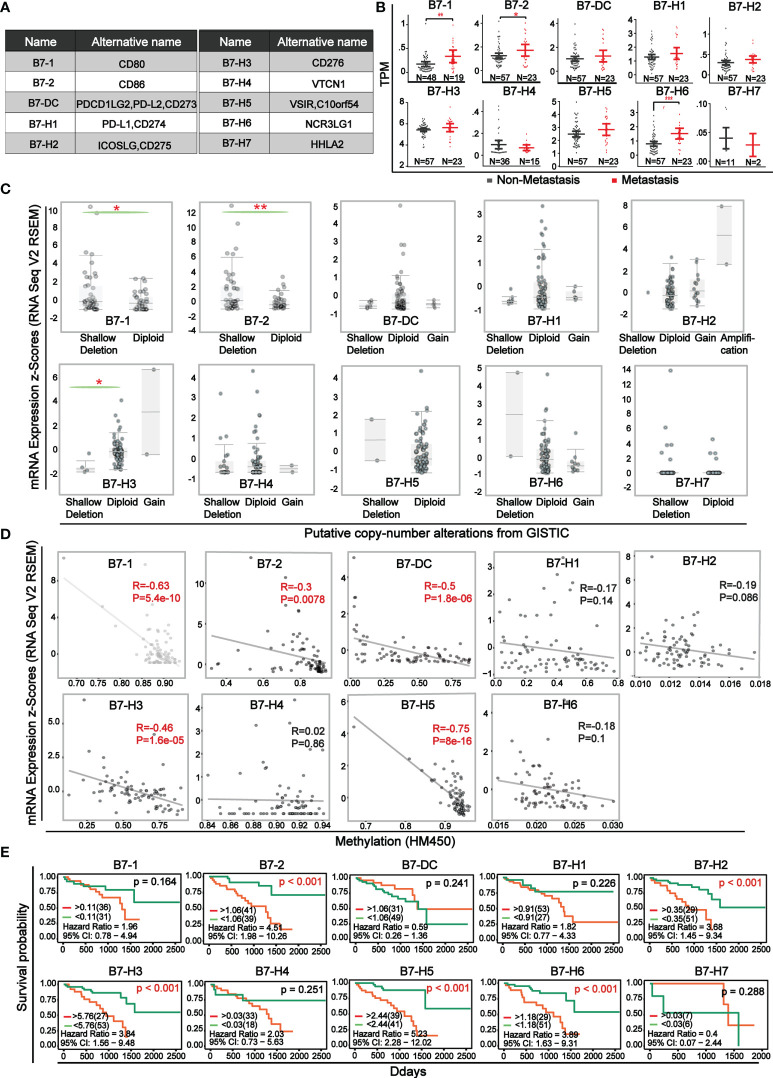
Expression level and survival curve of B7 family in UVM **(A)** Multiple denominate names of each B7 family member. **(B)** The comparison of mRNA expression of B7 family members between non-metastatic and metastatic UVM. Expression data and clinical information were from the TCGA database. N represented the sample size. **(C)** Association of B7 family member mRNA expression with gene alteration. **(D)** Correlation of B7 family member mRNA expression with promotor methylation. **(E)** Kaplan-Meier survival curves of B7 family genes in UVM based on the expression level. (* P < 0.05**, P < 0.01, and *** P < 0.001).

The ten B7 family members, as co-stimulatory or co-suppressive molecules of immunity were shown in **Figure 1A**. The expression level of B7 family members between non-metastatic and metastatic UVM was compared (**Figure 1B**). Results showed that there was significant elevation in metastatic UVM in *B7-1, B7-2*, and *B7-H6*. Comparison of B7 family expression between UVM and normal samples was shown in **Supplementary Figure 1** and the data was downloaded from GEO database (GSE176345). Then, for further study of the mechanism of B7 family members’ dysregulation in UVM, we explored the relationship of their mRNA expression level with gene alteration (shallow deletion, diploid, copy number gain, and gene amplification) and DNA methylation (**Figure 1C, D**). Gene alterations were associated with the expression of *B7-1, B7-2*, and *B7-H3* (**Figure 1C**). Moreover, there was a negative correlation between promotor methylation and mRNA expression for *B7-1, B7-2, B7-DC, B7-H3, B7-H5* (**Figure 1D**), suggesting that promotor methylation might also be involved in B7 members’ dysregulation. Then, we combined clinical information and gene expression data from the TCGA database to evaluate the effect of B7 expression on the survival rate of UVM patients. Kaplan-Meier analysis showed that the low expression of *B7-2, B7-H2, B7-H3, B7-H5, B7-H6* was significantly associated with longer OS in UVM patients (**Figure 1E**).

### Relationship between B7 family expression and infiltrating stromal and immune cells

**Figure 2:**
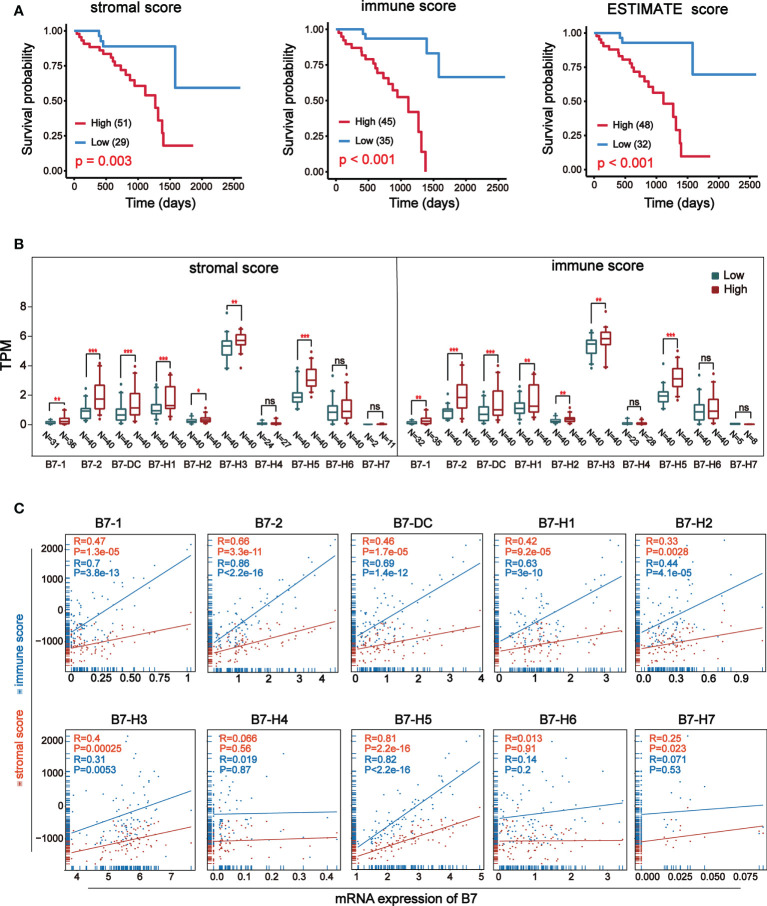
Relationship between stromal and immune score and expression of B7 family. Stromal and immune scores were obtained using expression data of UVM from the TCGA database. **(A)** Kaplan-Meier survival curves of ESTIMATE score. **(B)** The expression of B7 family was compared according to high and low score in stromal or immune score by median. The blue boxes represent low score, and the red boxes represent high score. annotation: ‘ns’: none significance. (* P < 0.05**, P < 0.01, and *** P < 0.001). **(C)** The correlation betweenB7 expression and stromal score and immune score.

ESTIMATE (Estimation of Stromal and Immune cells in Malignant Tumor tissues using Expression data) is a new algorithm to measure tumor cellularity and different infiltrating normal cells by transcriptional profile (31). Based on specific gene expression signatures of stromal and immune cells, we used stromal and immune scores to predict the level of infiltrating stromal and immune cells. First, we downloaded information of 80 UVM samples from TCGA database and got stromal and immune scores through the algorithm of ESTIMATE. The stromal score was between -1767.77 and -51.50 and the immune score was between -1172.97 and 2149.71. Prognosis analysis of ESTIMATE score, stromal score and immune score indicated that higher immune and stromal scores were significantly associated with worse prognosis (**Figure 2A**). Then, the expression of B7 family Was compared by dividing stromal score and immune score into high and low groups (**Figure 2B**). Results revealed that the expression of *B7-1, B7-2, B7-DC, B7-H1, B7-H2, B7-H3* and *B7-H5* was significantly higher in the high score group both in stromal and immune score. Next, we analyzed the correlation between B7 family expression and immune score and stromal score (**Figure 2C**). Consistent with the result in **Figures 2A**, **B**, the expression of *B7-1, B7-2, B7-DC, B7-H1, B7-H2, B7-H3 and B7-H5* was significantly and positively correlated with both stromal score and immune score.

**Figure 3:**
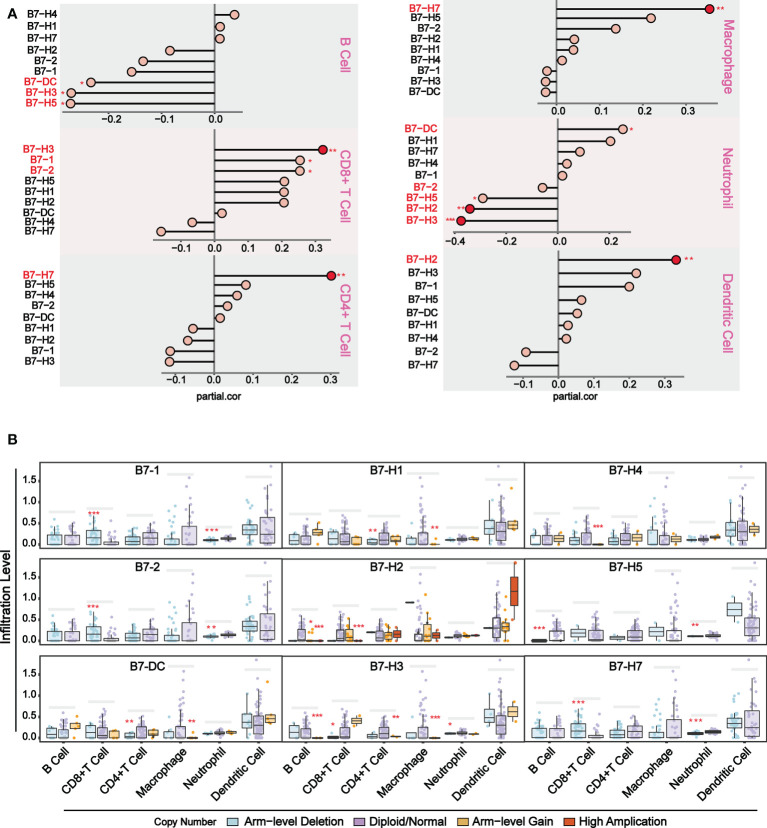
Association of immunologic infiltration with B7 family. **(A)** Correlation between B7 expression and infiltration level of immune infiltrating fluid cells. **(B)** Association of immune cell infiltration level with B7 family somatic copy number alterations. The infiltration abundance in every somatic copy number alteration category was compared to the diploid/normal. (* P < 0.05**, P < 0.01, and *** P < 0.001).

To further understand the relationship between B7 family expression and infiltrating immune cell type, we analyzed six tumor-infiltrating immune cell types (B cells, CD4 T cells, CD8 T cells, neutrophils, macrophages, and dendritic cells) in the immune microenvironment. Using TIMER (https://cistrome.shinyapps.io/timer/), we analyzed the correlation between gene expression of B7 family and the infiltration level of immune cells in the immune infiltration fluid and compared the abundance of tumor-infiltrating immune cells with different somatic copy number aberrations for B7 family (**Figure 3**). As shown in **Figure 3A**, results revealed that the expression of *B7-1* and *B7-2* showed a weak positive correlation with CD8+ T cell infiltration level, and the expression of *B7-DC* and *B7-H5* has a weak positive correlation with neutrophils and a weak negative correlation with B cell infiltration level. *B7-H2* expression is significantly negatively related to neutrophils and has significant positive correlations with dendritic cells infiltration level. *B7-H3* expression showed significantly negatively related to neutrophils and has significant positive correlations with CD8+ T cells but weak positive correlation with B cells infiltration level. The expression of B7-H7 is significantly positively related to CD4+ T cells and macrophages infiltration level. Furthermore, we compared immune cell infiltration level with different somatic copy number alterations of B7 family in UVM (**Figure 3B**). Alteration in *B7-1, B7-2, B7-H2, B7-H3, B7-H4* and *B7-H7* significantly associated with the infiltration levels of CD8+ T cells and *B7-H2, B7-H3* and *B7-H5* significantly associated with the infiltration levels of B cells. *B7-DC, B7-H1, B7-H3* significantly associated with the infiltration levels of CD4+ T cells and macrophages. *B7-1, B7-2, B7-H5, B7-H5, B7-H7* significantly associated with the infiltration levels of neutrophils.

### Immune cell composition in UVM and its relationship with B7 family expression

**Figure 4:**
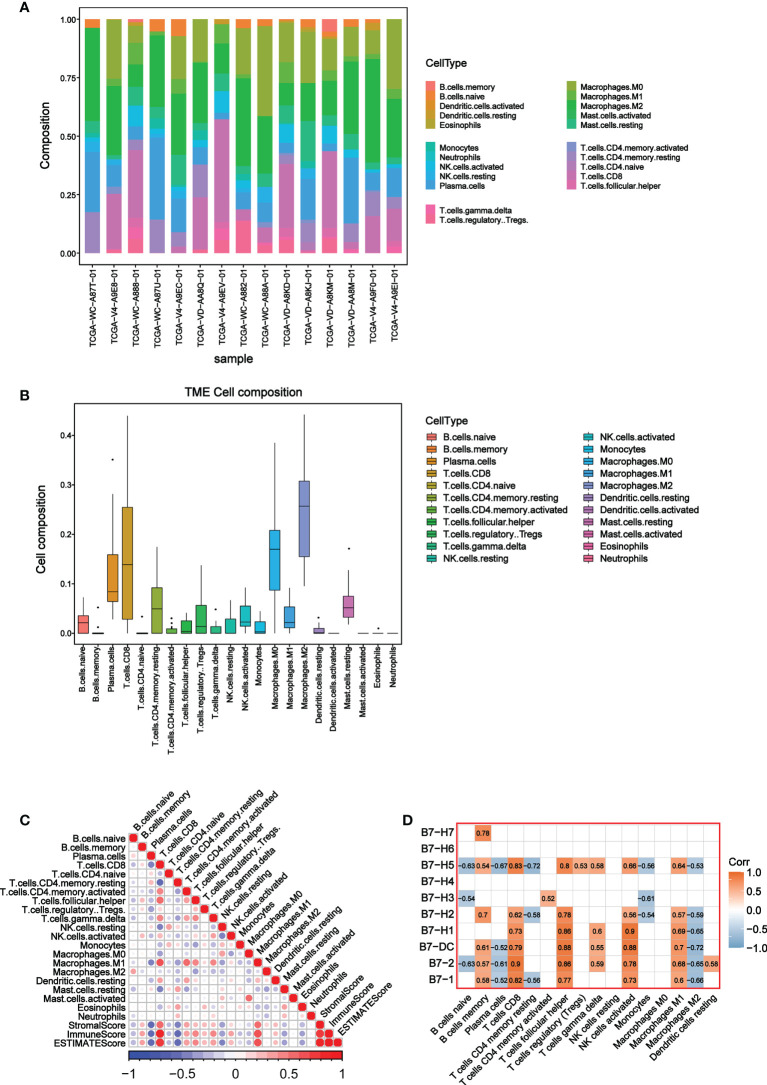
Correlation between B7 family and CIBERSORT score. **(A)** Cell composition in each UVM sample. **(B)** Composition of 22 immune-related cells in TME. **(C)** Correlation analysis among 22 immune-related cells, stromal score, immune score and ESTIMATE score. **(D)** Correlation analysis between 22 immune-related cells and B7 family.

To further explore the relationship between B7 family and TME, we conducted the CIBERSORT analysis (a method for characterizing cell composition of complex tissues from their gene expression profiles) (29). First, we evaluated the composition of 22 immune-related cell types in each UVM sample (**Figure 4A**) and their cellular composition in the TME (**Figure 4B**). The results showed that different sample had varied TME composition and the main cell composition were macrophages, NK cells, and T cells. In addition, we analyzed correlations between 22 immune cells and estimate score, which revealed they have a significant correlation (**Figure 4C**). High immune responses were positively correlated with CD8+ T cells and M1 macrophages. Further analysis of the association of the B7 family and 22 immune cells demonstrated that the expression of the B7 family was significantly correlated with immune cells, especially positively correlated with CD8+ T cells, M1 macrophages, T helper cells and activated NK cells while negatively correlated with M2 macrophages (**Figure 4D**). Thus, we conclude that expression of the B7 family may participant in the immune response of different immune cells, thus affecting the TME in UVM.

### Single cell sequencing analysis further reveals the correlation of B7 family with immune cell types

**Figure 5:**
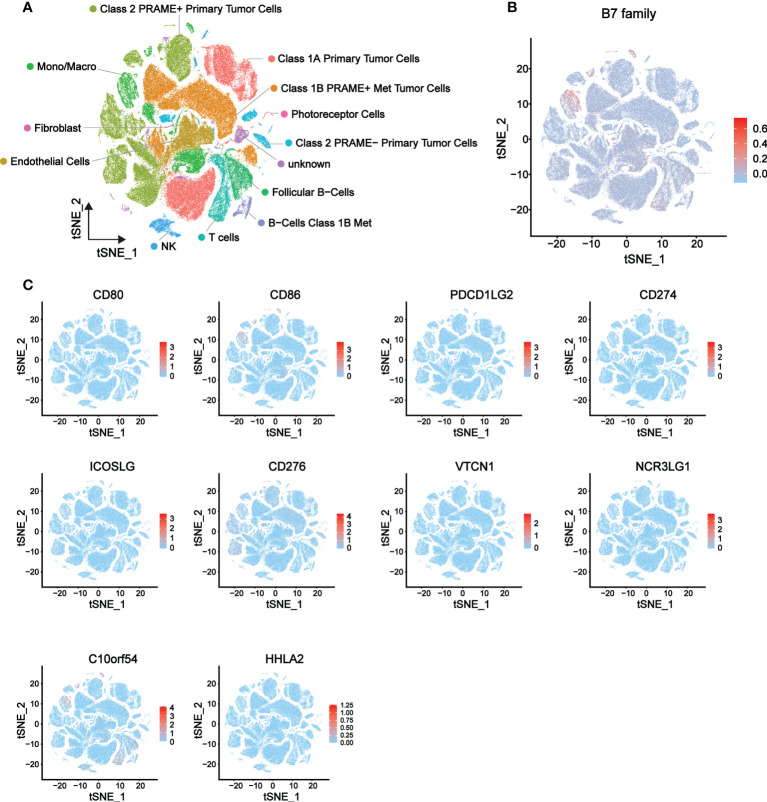
Expression distribution of the B7 family in eight primary and three metastatic UVMs from GSE139829 dataset. **(A)** 13 cell type distribution and the cell type annotations. **(B)** The overall expression distribution of the B7 family in 13 cell types. **(C)** The respective expression distribution of the B7 family in 13 cell types.

To further explore the relationship of B7 family expression with the TME of UVM, we analyzed single-cell sequencing data (a total of 171941 cells) from 3 metastatic and 8 orthotopic tumor samples in the GSE139829 data set, and the expression distribution of B7 family in different cells of UVM was described. The overall distribution of the 13 cell types is shown in **Figure 5A** and related data shown in supplementary **Figure 2**. Results showed that B7 family was mainly expressed in immune cells, such as monocytes/macrophages, NK cells, T cells, and B cells (**Figure 5B**). Further specific analysis of the expression of each member of the B7 family showed that the expression of B7-2, B7-H3 and B7-H5 were significant (**Figure 5C**). Among them, B7-2 was specifically distributed in macrophages, B7-H5 was mainly distributed in macrophages, NK cells, T cells and B cells, while B7-H3 was mainly distributed in malignant tumor cells. Thus, these results suggested that the B7 family might be involved in TME reconstitution by influencing immune cells and malignant tumor cells.

### Function and pathway analysis of B7 family members

**Figure 6:**
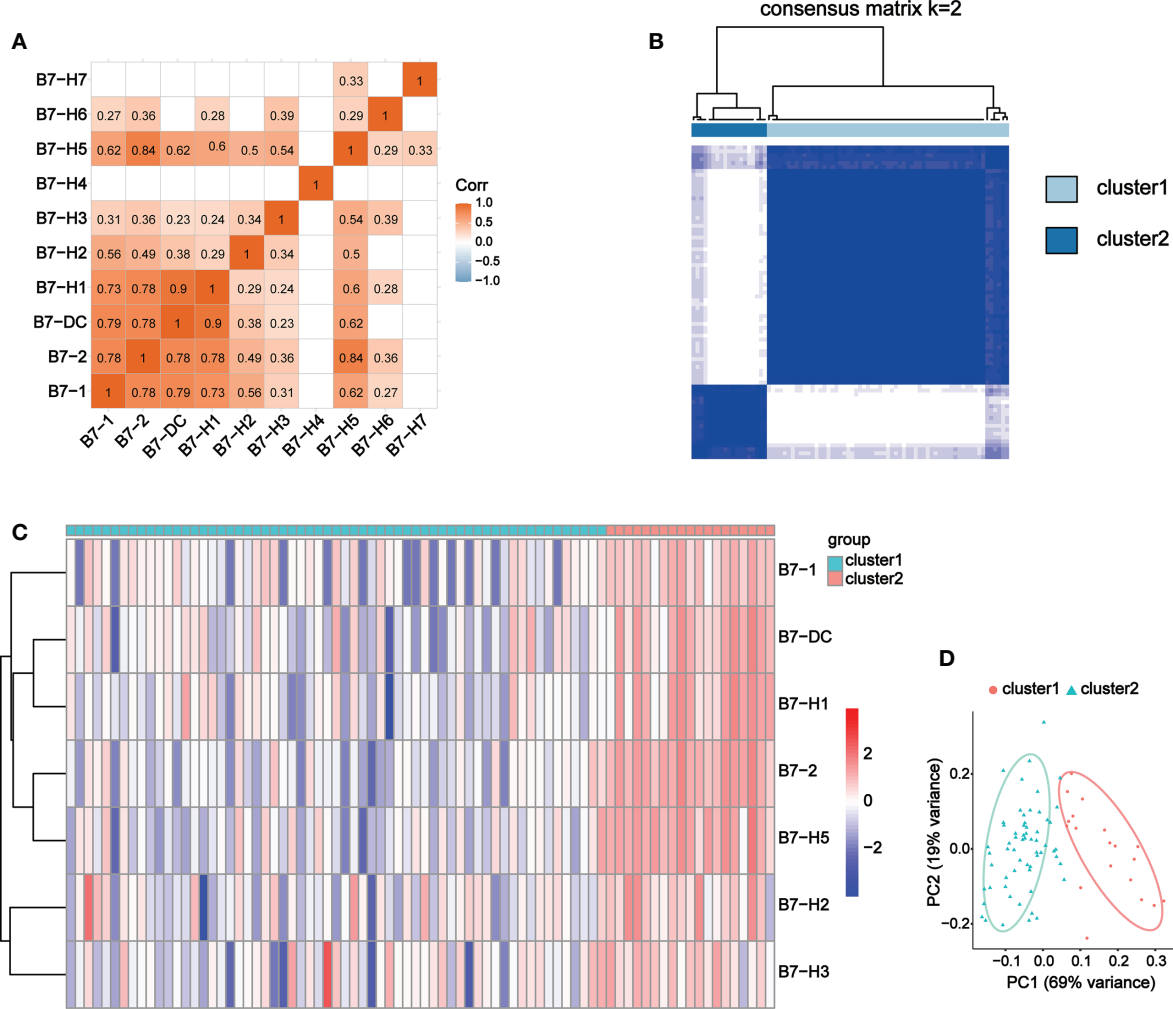
Co-expression analysis of B7 family. **(A)** Correlation analysis of B7 family. **(B)** Consensus clustering matrix for k = 2. **(C)** Expression heatmap of B7 family grouped by consistency of k = 2. **(D)** Principal component analysis (PCA) of 2 subtypes based on high and low expression of the B7 family.

We conducted correlation analysis and obtained 8 B7 family members with significant correlation, namely B7-1, B7-2, B7-DC, B7-H1, B7-H2, B7-H3, B7-H5 and B7-H6 (**Figure 6A**). Due to the weak correlation between B7-H6 and immunity and the other family members, we excluded it from subsequent clustering analysis. 80 UVM samples were divided into two groups according to expression level of these B7 members through consistency cluster analysis (**Figure 6B**). One group contained 19 high-expression samples and the other consisted of 61 low-expression samples. The expression heatmaps of the two groups were shown in **Figure 6C**. In addition, principal component analysis (PCA) was performed and the results showed that the high and low expression of B7 family can distinguish UVM patients (**Figure 6D**).

**Figure 7:**
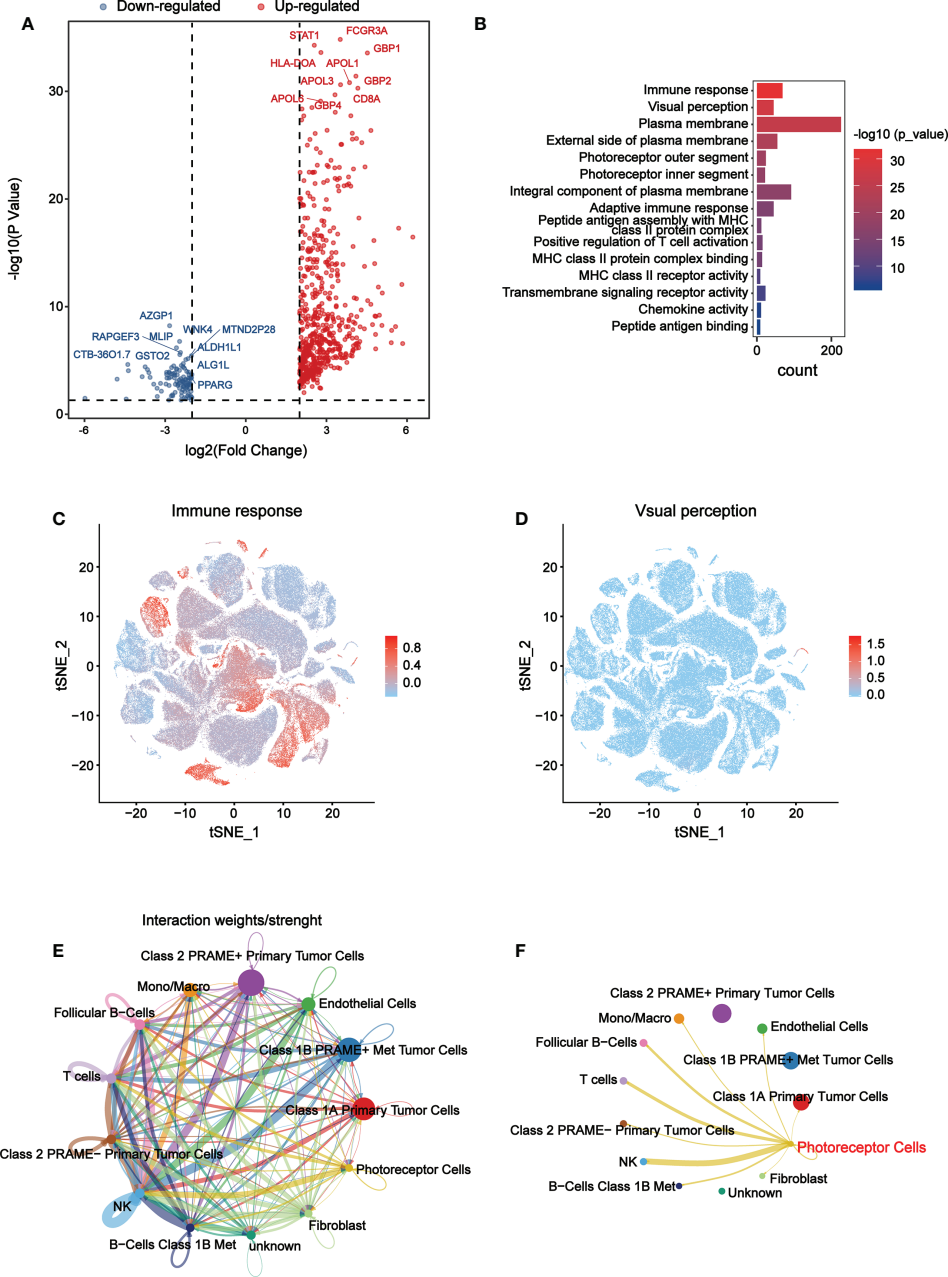
Main functions affected by B7 family. **(A)** Volcano plot was drawn to identify DEGs affected by B7 members. The Y axis is the value of fold change of expression level that based on the logarithmic ratio (mean of changed expression/mean of unchanged expression). -log10 p-value > 1.3 and log ratio > 2 or log ratio< -2 is considered to be significantly different. **(B)** The most significant functions were shown according to P value and gene count. The overall expression distribution of DEGs in immune response function **(C)** and visual perception function **(D)** in 13 cell types. **(E)** An overview of cell-cell interactions in 13 cell types. Arrow and edge color indicate direction. Edge thickness indicates the weights/strength of interaction between cells. **(F)** cell-cell interactions map between photoreceptor cells and other 12 cell types.

To explore the function and signaling pathways of differentially expressed genes (DEGs) in the B7 family, KEGG and GO enrichment analyses were performed. First, the DEGs were identified by the high and low expression level of B7 family according to consistency cluster analysis. The volcano map showed significant DEGs (FDR<0.05) for further analysis (**Figure 7A**). We used DAVID website to enrich the functions of the DEGs in B7 family. The upregulated DEGs of the B7 family primarily affect immune response and visual perception function (**Figure 7B**). KEGG pathway enrichment analysis revealed that the up-regulated DEGs mainly affected immune-related pathways, such as Th1 and Th2 cell differentiation, Th17 cell differentiation and primary immunodeficiency (**Supplementary Figure 3A**). Down-regulated DEGs of the B7 family mainly affected positive regulation of cell death function and glutathlone metabolism pathways (**Supplementary Figures 3B, C**).

Next, we analyzed the overall distribution of DEGs in immune response function and visual perception function in 13 cell types using scRNA-seq data. As shown in **Figures 7C, D**, genes enriched in immune function were mainly expressed in macrophages, NK cells, T cells and B cells, while genes enriched in visual perception function were mainly expressed in photoreceptor cells. CellChat is used for complex cell-to-cell communication analysis. Cell Chat analysis was used to further explore the interaction relationship between 13 cell types (**Figures 7E, F** ***)*** and results showed that photoreceptor cells had significant interaction with NK cells (**Figure 7F**).

### Target gene analysis of B7 family members

**Figure 8:**
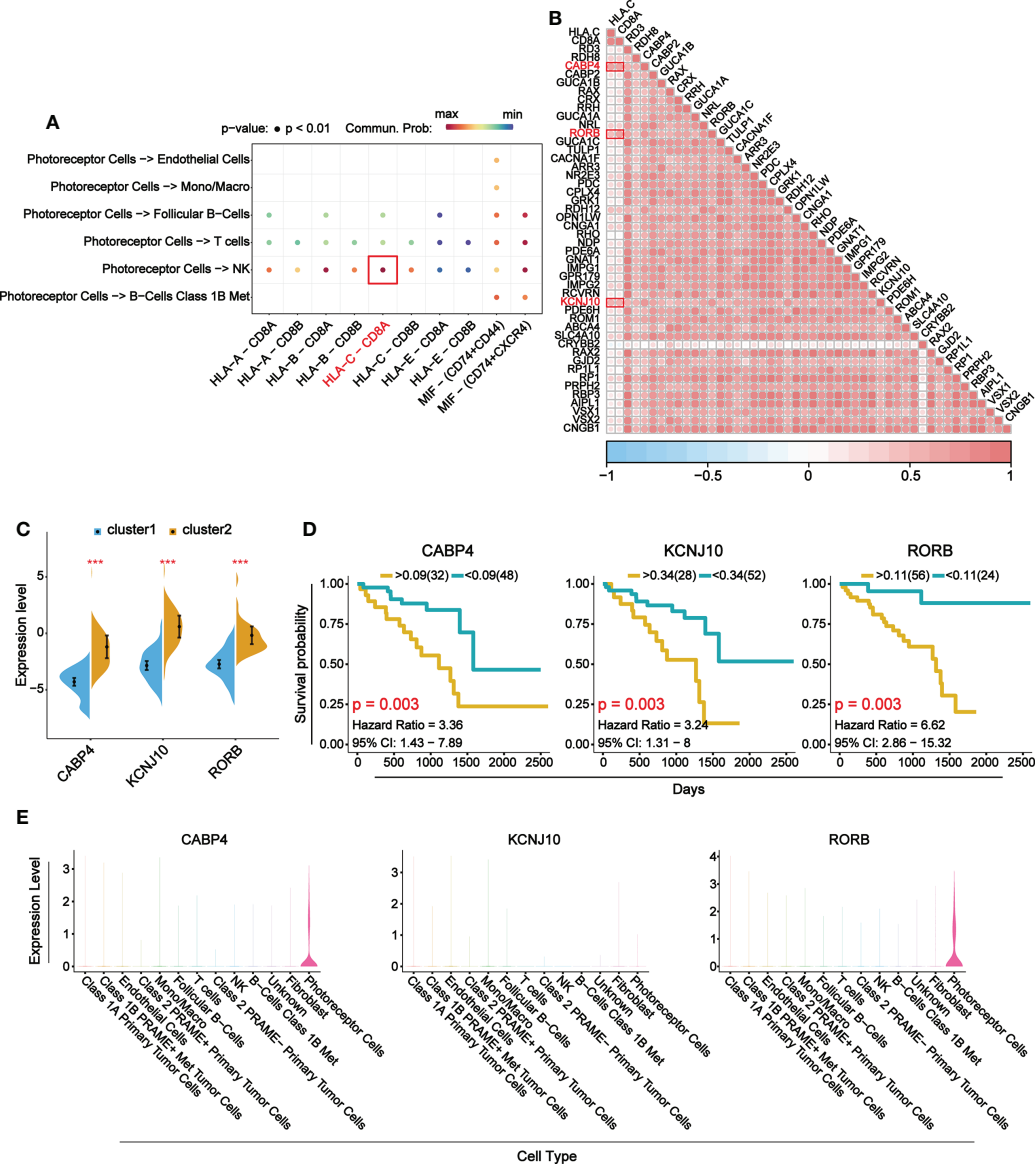
Main target genes of B7 family. **(A)** Receptor ligand pairs in which photoreceptor cells interact with immune-related cells. **(B)** Correlation analysis between receptor-ligand pair genes and DEGs in visual perception function. **(C)** The comparison of mRNA expression of top 3 DEGs between high and low expression group of B7 family. **(D)** Prognosis analysis of top 3 DEGs. **(E)** Violin plots show the expression levels of the top 3 DEGs in 13 cell types. *P < 0.05, **P < 0.01, and ***P < 0.001.

To explore the main target gene of B7 family in UVM, we determined the receptor ligand pair with the strongest interaction between immune-related cells and photoreceptor cells and found HLA-C-CD8A (**Figure 8A**). The correlation analysis of HLA, CD8A and DEGs enriched in visual perception function showed that three target genes with the strongest correlation were CABP4, KCNJ10 and RORB (**Figure 8B**). Further differential expression analysis and prognosis analysis of these three target genes showed that they were differentially expressed in UVM and their high expression was associated with poor prognosis, which was consistent with B7 family (**Figures 8C, D** ***)***. Finally, expression distribution analysis showed that CABP4 and RORB gene were specifically expressed in photoreceptor cells (**Figure 8E**). Therefore, CABP4 and RORB gene may be the main potential target gene of B7 family in UVM.

## Discussion

Although UVM is an uncommon tumor, its impact on the eyes and lethality prompt us to study its mechanism and potential therapeutic target. About 50% of all UVM patients will have metastatic disease, mainly in liver. There are several local treatment options at present, but none of these strategies has provided survival benefit for patients with hepatic metastatic UVM (32). Currently, there is no standard treatment for metastatic UVM. Immunotherapy as a potential treatment has been studied in UVM (33). Immune checkpoint inhibitor (ICI) is a hot topic in the immunotherapy of various types of cancer in recent years. Cytotoxic T lymphocyte antigen 4 (CTL-4) and programmed cell death protein 1 (PD-1)/programmed cell death-ligand 1 (PD-L1) are representative ICI s and antibodies against them have been widely approved by the FDA for cancer treatment (34). Cutaneous melanoma (CM) is the first tumor treated with immune checkpoint inhibitors approved by PDA, and its treatment has achieved excellent results (35). However, in UVM, the response to ICIs is disappointing (33). Therefore, controlling the local tumor, reducing the risk of metastasis, protecting the eye and maintaining visual function are the main goals of UVM treatment. Low mutational burden, poor immunogenicity and low expression of PD-L1 may be the direct factors leading to the poor response of UVM immunotherapy (17, 36). B7 family members, as immune stimulator or suppressor molecules, participate in immune checkpoint and tumor angiogenesis, and play an important role in the development of malignant tumors (8). However, there are few studies of B7 family in UVM, and only a few members have been mentioned, such as *B7-1, B7-DC, B7-H1, B7-H3* and *B7-H4* (18, 37). The expression pattern, the effect on immune cells and TME and the downstream signaling pathway of B7 family in UVM have not been well elucidated. Therefore, we conducted bioinformatics analysis of B7 family members in UVM.

In this study, we first obtained the expression profile and clinical data from TCGA, and analyzed the expression of B7 family in non-metastatic and metastatic UVM. The results showed that the expression of *B7-1, B7-2* and *B7-H6* in metastatic UVM was significantly higher than that in non-metastatic UVM (**Figure 1B**), indicating that increased expression of *B7-1, B7-2* and *B7-H6* may promote metastasis of UVM. Studies have shown that *B7-1* may play an important role in regulating the development and metastasis of gastric cancer (38, 39). High expression of *B7-1* and *B7-2* may indirectly affect the lymph node metastasis of colorectal cancer by influencing the expression of CD14 + macrophages (40). *B7-H6* expression also affects the invasion and metastasis of a variety of tumors, such as glioma cells, ovarian cancer (41, 42). There is still lack of research on the effect of *B7-1, B7-2* and *B7-H6* on metastasis of UVM. Next, we further analyzed the dysregulation mechanisms of B7 family. By determining the association of genetic alteration and DNA methylation with gene expression, we found that genetic alteration (shallow deletion, diploid) in *B7-1, B7-2, B7-H3* affected their expression and DNA methylation in *B7-1, B7-2, B7-DC, B7-H3, B7-H5* played a part in their dysregulation (**Figures 1 C, D**). In order to explore whether the expression of B7 family members was an independent prognostic factor for UVM, Kaplan- Meier analysis was conducted. The results showed that the higher expression of *B7-2, B7-H2, B7-H3, B7-H5, B7-H6* was significantly correlated with poor overall survival (OS) (**Figure 1E**). Previous studies have shown that high expression of *B7-2, B7-H2, B7-H3, B7-H5, B7-H6* were also associated with poor prognosis in other cancers, which is consistent with our results (43–46).

The TME has been shown to be extensively involved in tumorigenesis because it contains tumor cells that interact with surrounding cells through the lymphatic and circulatory systems, thereby influencing cancer development and progression. In addition, non-malignant cells in the TME play a critical role in all stages of carcinogenesis by stimulating and promoting uncontrolled cell proliferation (47). Among the immune checkpoint molecules, B7 family is significantly involved in immune escape of tumor cells, which exists in different stages of TME formation and promotes tumorigenesis and tumor progression (48). B7 family has been found to affect the formation of tumor immune microenvironment in a variety of cancers (49). For example, B7-H3 and B7-H4, as co-regulatory ligands in B7 family, play a negative role in cervical cancer microenvironment by regulating the expression of IL-10 and TGF-β1 (14). Till now, the association of B7 family on the formation of UVM TME remains unknown.

In order to explore the relationship between B7 family and TME formation, we first analyzed the relationship between B7 family expression and infiltrating stromal and immune cells. Infiltrating stromal and immune cells constitute the main part of normal cells in tumor tissues, which not only interfere with tumor signals in molecular research, but also play an important role in cancer biology (31). Stromal cells are thought to have important roles in tumor growth, disease progression, and drug resistance (50–52). Infiltrating immune cells act in a context-dependent manner and affects treatment and prognosis (53). We calculated stromal and immune score by the ESTIMATE algorithm and found that higher immune and stromal scores were significantly associated with worse prognosis (**Figure 2A**). The immune score was higher than stromal score. As stromal scores and immune scores were generated to reflect the presence of stromal and immune cells respectively (54), we concluded immune cells may have a greater effect on UVM than stromal cells. By further studying the effect of B7 family on stromal cells and immune cells, we found that high expression of *B7-1, B7-2, B7-DC, B7-H1, B7-H2, B7-H3, B7-H5* were significantly and positively correlated with high stromal and immune score (**Figure 2B, C**), suggesting that the high expression of B7 family was associated with stromal and immune cell infiltration in UVM. Besides, the expression of B7 family was more significantly correlated with immune scores than stromal scores, indicating they had greater correlation immune cell infiltration in UVM.

In UVM, immune cell infiltration is a marker of poor prognosis (55). Increasing evidence revealed that B7 family has a great impact on immune modulation. For example, *CD80* expression can prevent PDL1-mediated immunosuppression of tumor cells and restore T cell activation (56). The expression of B7-1 negatively regulates T cell immune responses by inhibiting T cell activation rather than by promoting T cell apoptosis (19). The molecules *B7-1* and *B7-2* together stimulate T cells to mediate antitumor immunity (39). Previous studies have found that some B7 family members have an effect on immune infiltration, such as *B7-H1* and *B7-H4* (57, 58), but there has been no comprehensive study of the effect of B7 family members on immune infiltration in UVM. To further understand the relationship between B7 family and immune cell infiltration in UVM, we explored the correlation between the expression of B7 members and the infiltration levels of different immune cell types (**Figure 3A, B**). We observed that B cell, CD8^+^ T cell and neutrophil were the main immune cells affected by B7 family. B cell mainly had negative correlation and CD8^+^ T cell had positive correlation with B7 expression. Moreover, the expression of *B7-DC, B7-H2, B7-H3, B7-H5* and *B7-H7* were significantly associated with more immune cell type than other B7 members. Furthermore, we compared infiltration levels with different somatic copy number alterations for B7 family in UVM (**Figure 3B**). We found that somatic changes in the B7 family also have an effect on immunocyte infiltration.

By CIBORSORT analysis, we further explored the relationship between B7 family and TME cells in UVM. Recent study has similarly demonstrated that B7-H3-rich tumors were rich in macrophages M1, CD8+ T cells and NK cells in rhabdomyosarcoma (59). Significantly higher CD8 + T cell infiltration and enrichment of CD56bright NK cells were found when PD-L1 was highly expressed in the non-small-cell lung carcinoma TME (60). However, low expression of PD-L1 significantly increased the expression of CD4 + T cells, CD8 + T cells, NK cells and CD11C + M1 macrophages in ovarian cancer, whereas significantly inhibited the expression of regulatory T cells (61). In UVM, we found most members of B7 family were significantly correlated with immune cells, especially macrophages, NK cells and T cells (**Figures 4A–D**). In particular, the high expression of B7 family may promote the immune response of macrophages M1, NK cells and CD8+ T cells, thereby affecting the TME of UVM. Single-cell sequencing analysis also revealed that the B7 family is generally abundant in monocytes/macrophages, NK cells, T cells, and B cells (**Figures 5A–C**). Interestingly, B7-2 is specifically distributed in macrophages. By combining multiple analyses, we conclude that the B7 family plays a significant role in UVM, primarily by influencing immune cells to modulate the TME.

At present, studies of UVM have elucidated many mechanisms and found new predictors and potential targets (62–64), but no specific mechanism of the B7 family has been studied. To explore the mechanism of B7 family, we performed GO and KEGG analysis. We found DEGs affected by B7 family mainly play an important role in the regulation of immune response function, visual perception function and immune-related pathways (**Figures 7A, B**; **Supplementary Figure 3**). Furthermore, genes enriched in immune response function were found to be mainly expressed in macrophages, NK cells, T cells and B cells, whereas genes enriched in visual perception function were mainly expressed in photoreceptor cells (**Figures 7C, D**). Immune-related genes interact significantly with photoreceptors, especially NK cells (**Figure 7F**). We hypothesize that the B7 family affects the function of photoreceptors by influencing the actions of immune-related cells in UVM. Studies have shown that cutaneous malignant melanoma or uveal melanoma can lead to melanoma-associated retinopathy (Mar) (65, 66). Mar is a paraneoplastic syndrome in which anti-retinal antibodies cross-react with retinal bipolar cells, leading to night blindness and progressive loss of field of vision (67). Current studies have shown that microglia and immunity are associated with loss of photoreceptors in the retina (68). Infiltration of microglia/macrophages and upregulation of cytokines are related to apoptosis and regulated necrosis of photoreceptors in Retinitis pigmentosa (69, 70). Activated microglia, macrophages and Müller glia can release inflammatory factors, such as TNF α, leading to apoptosis or necroptosis in photoreceptors (71, 72). In addition, IFN-γ and IL-17A of specific T cells in mice with autoimmune uveitis have cytotoxic effects on photoreceptor cell proliferation (73). T cells may play a major role in the pathology of retinal choroiditis (74). However, the mechanism of interaction between immune cells and photoreceptors remains unclear in UVM. Next, the receptor ligand pair HLA-C-CD8A with the strongest interaction between NK cells and photoreceptor cells were obtained (**Figure 8A**). This receptor ligand pair mainly plays a role in cell adhesion molecules pathway. Finally, by correlation analysis, differential expression analysis, prognostic analysis and expression distribution analysis, the main potential target genes of B7 family, which are CABP4 and RORB, were identified (**Figures 7B–E**). CABP4, a member of a sub-family of neuronal Ca2+-binding proteins that are highly similar to calmodulin, is located at the synaptic end of photoreceptors and is required for normal neurotransmission between photoreceptors and bipolar cells (75). CABP4 is associated with retinopathy and plays an important role in visual perception (76, 77). RORB exists in immature neurons and is hypothesized to play a role in neuronal differentiation. RORB has two different isoforms, RORβ1 and RORβ2, and RORβ2 is mainly expressed in retina and pineal gland (78). Recently, studies have found that these two genes play an important regulatory role in the development and progression of various cancers (79–81). CABP4 has been reported to be associated with the TME of urothelial carcinoma of the bladder currently (79). RORB can down-regulate the activity of Wnt signaling pathway, thus inhibiting the stemness of gastric cancer cells (82). RORB has also been found to be a prognostic marker of breast cancer (83). In addition, we found that CABP4 and RORB have a significant prognostic impact in UVM. We hypothesize that CABP4 and RORB might be involved in the interaction of NK cells and photoreceptor cells, which can be influenced by B7 expression and might participate in symptoms associated with UVM, like retinopathy or loss of photoreceptor. Thus, targeting these genes might possibly alleviated these symptoms. Our analysis reveals the important role of the CABP4 and RORB genes in UVM for the first time. They may hold more therapeutic promise than the weak effects of ICIs.

## Conclusions

As far as we know, this is the first systematic study on the expression pattern, immune cell infiltration, downstream signaling pathway and hub genes of B7 family members in UVM. Dysregulation of the B7 family in UVM is associated with poor prognosis and affects the tumor immune microenvironment. In particular, we found that *B7-2*, which can serve as a marker for macrophages in UVM, had a significant effect on the metastasis, prognosis and immunocyte infiltration of UVM through bioinformatics analysis. Thus, it may be important in the development and progression of UVM. Moreover, CABP4 and RORB can serve as potential therapeutic targets for UVM, which are regulated by the B7 family to affect the visual perception and immune response function of the eye, thus influencing the prognosis of UVM. In general, our findings provide new insights into the role of B7 family members in UVM, and may be of great significance for immunotherapy of UVM in the future.

## Data availability statement

The datasets presented in this study can be found in online repositories. The names of the repository/repositories and accession number(s) can be found within the article/supplementary materials.

## Author contributions

Conceptualization and methodology: YuC, AFZ; Formal analysis and investigation: YuC, AFZ, YaZ, MTX; Writing original Draft preparation: YaC, AFZ, YZ YuZ, XW, MXL; Writing review and editing: JS, ZGX, FKD, YuC, MJC, WPL, XBL, YHS, LG. All authors contributed to manuscript revision, read, and approved the submitted version.

## Funding

This work was supported by National Natural Science Foundation of China (No. 81972643, No. 82172962) and Sichuan Science and Technology Project (2021YJ0201).

## Acknowledgments

Thanks to YZ and MX for their help in analysis, and to JS, ZX, YsZ, XW, ML, FD, YC, MC, WL, XL, YS, and LG for their help in writing.

## Conflict of interest

The authors declare that the research was conducted in the absence of any commercial or financial relationships that could be construed as a potential conflict of interest.

## Publisher’s note

All claims expressed in this article are solely those of the authors and do not necessarily represent those of their affiliated organizations, or those of the publisher, the editors and the reviewers. Any product that may be evaluated in this article, or claim that may be made by its manufacturer, is not guaranteed or endorsed by the publisher.
