# Supplementary material for: Dysregulation of B7 family and its association with tumor microenvironment in uveal melanoma
Source: Front Immunol. 2022 Oct 14;13:1026076. doi: 10.3389/fimmu.2022.1026076 (PMC9615147; doi:10.3389/fimmu.2022.1026076)
Supplement: Supplementary file 1 [file DataSheet_1.pdf]

Supplementary figure

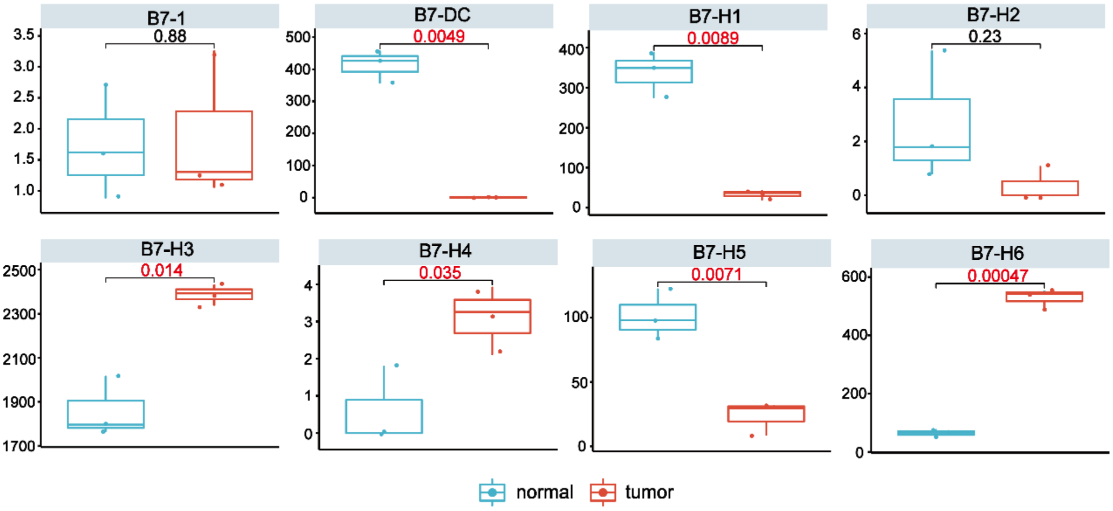

Supplementary figure 1 Comparison of the expression levels of B7 family members in normal and tumor samples of UVM.

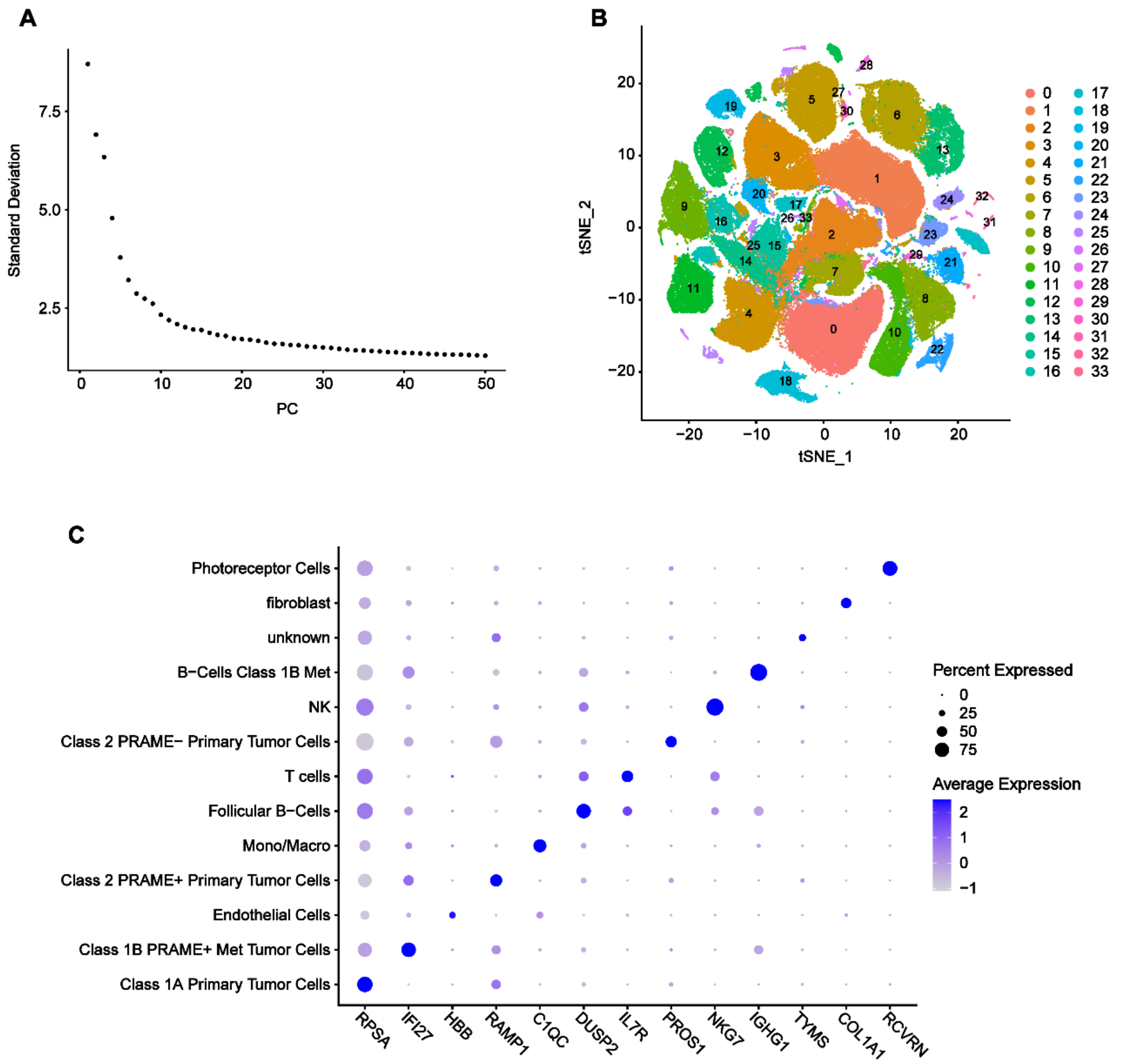

Supplementary figure 2 Expression levels of key genes in UVM cells. (A) ElbowPlot to determine the dimensionality of the data set. The top 30 PCs can explain most of the variation. (B) Clustering of single cells in TSNE space, showing 34 clusters (clusters 0-33). (C) Bubble plots show the expression levels of marker genes in 13 cell types.

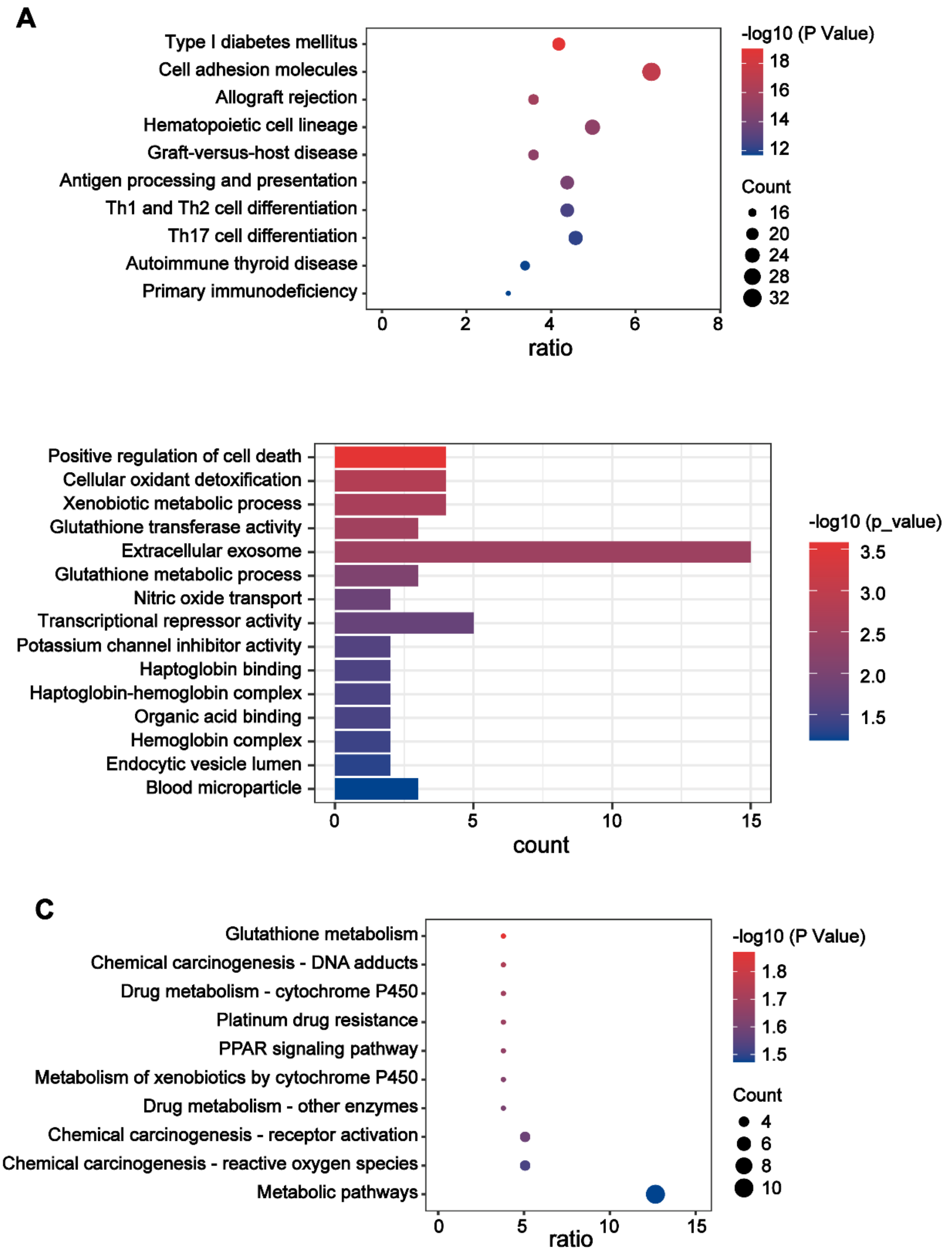

Supplementary figure 3 Main signaling pathways and functions affected by B7 family members. (A) KEGG pathway enrichment analysis of the up-regulated DEGs. (B) GO analysis of the down-regulated DEGs. P values and gene count were considered. (C) KEGG pathway enrichment analysis of the down-regulated DEGs.
